# Sertraline plus repetitive transcranial magnetic stimulation for the treatment of postpartum depression

**DOI:** 10.1590/1806-9282.20241001

**Published:** 2025-06-02

**Authors:** Zhengyu Zhang, Ting Luo, Jianying Yu

**Affiliations:** 1 Sichuan University, West China Hospital, Mental Health Center – Chengdu, China.; 2 Sichuan University, West China Hospital, Department of Radiology – Chengdu, China.

**Keywords:** Postpartum depression, Magnetic stimulation, transcranial, Sertraline, Quality of life, Norepinephrine, serotonin, Estrogen

## Abstract

**OBJECTIVE:**

Postpartum depression significantly affects maternal mental health and family dynamics. Effective and safe treatments are crucial to enhance the quality of life for affected women. This study assesses the effectiveness and safety of combining sertraline with repetitive transcranial magnetic stimulation in treating postpartum depression.

**METHODS:**

We recruited 152 postpartum depression patients at West China Hospital between May 2020 and October 2023, dividing them into sertraline-only (n=61) and combined treatment (n=91) groups. We evaluated treatment outcomes after 8 weeks, comparing effectiveness, quality of life, neurotransmitter levels, and estrogen levels, while monitoring for adverse reactions.

**RESULTS:**

After 8 weeks, the combined treatment group exhibited a significantly higher effectiveness rate (95.6%) compared to the sertraline-only group (78.7%; p=0.001). Factors such as age, marital status, and treatment regimen significantly influenced treatment outcomes, with women under 30 and married women showing superior results. Post-treatment, both groups showed improvements in physical, psychological, social, and environmental aspects of life, with the combined group achieving notably higher scores. Improvements in neurotransmitter levels (5-hydroxytryptamine and plasma norepinephrine) and hormonal balances (luteinizing hormone, follicle-stimulating hormone, progesterone, and estradiol) were more substantial in the combined treatment group. Both groups had similar rates of adverse reactions, indicating that combining treatments did not significantly increase adverse events (11.0% in the combined group vs. 4.9% in the sertraline group; p=0.190).

**CONCLUSIONS:**

Combining sertraline with repetitive transcranial magnetic stimulation significantly enhances treatment effectiveness and improves neurotransmitter and hormone levels, contributing to better quality of life outcomes for postpartum depression patients without increasing adverse reactions.

## INTRODUCTION

Pharmacotherapy is commonly used in the management of postpartum depression (PPD), with selective serotonin reuptake inhibitors (SSRIs) being extensively utilized^1^. Sertraline, a specific SSRI, increases the availability of serotonin, which is crucial for mood regulation. Additionally, sertraline indirectly affects dopamine neurotransmission by modulating 5-HT2A receptors on the presynaptic membranes of dopamine neurons. Although sertraline is a first-line antidepressant, its effectiveness varies and often does not achieve full remission of symptoms in all patients. Furthermore, many women are hesitant to take medication during the postpartum period due to concerns about side effects and the potential impact on breastfeeding. This highlights the need to explore more effective and acceptable treatment modalities for PPD^2^.

Repetitive transcranial magnetic stimulation (rTMS) enhances the functionality of the brain's prefrontal cortex, playing a significant role in depression treatment^3^. rTMS is a non-invasive, painless method, offering high acceptability among patients. It has been validated for managing various forms of depression, including treatment-resistant cases. Additionally, rTMS has received FDA approval for the treatment of clinical depression, addressing limitations in pharmacotherapy and other physical treatments^4^. This study aims to assess the clinical effectiveness of combined rTMS and sertraline treatment for PPD, specifically examining its effects on neurotransmitter and estrogen levels.

## METHODS

### Patients

This retrospective study evaluated clinical data from patients diagnosed with PPD and treated at the Mental Health Center of West China Hospital, Sichuan University, between May 2020 and October 2023.

Patients received either sertraline monotherapy or a combination therapy of sertraline and rTMS. The inclusion criteria were (1) meeting the diagnostic criteria for PPD according to Chinese guidelines^5^; (2) Edinburgh Postnatal Depression Scale (EPDS) score greater than 13^6^; (3) full-term pregnancy; (4) absence of pregnancy complications; (5) favorable mother and infant outcomes; (6) onset of symptoms within 6 weeks postpartum; and (7) no prior related treatment. The exclusion criteria included (1) allergy to the study medication; (2) breastfeeding; (3) severe organic diseases; (4) history of depression or other psychiatric disorders; (5) concurrent endocrine dysfunction or central nervous system diseases; (6) history of brain surgery or suicidal tendencies; (7) acute or chronic systemic infectious diseases or immunodeficiency disorders; and (8) intolerance to electrostimulation therapy. All patients provided informed consent, and the study was approved by the Ethics Committee of West China Hospital, Sichuan University.

### Clinical treatment

Patients in both groups received foundational treatments, including psychotherapy and cognitive–behavioral interventions. In the sertraline group, patients were prescribed sertraline hydrochloride tablets, starting with a daily dose of 50 mg, which was gradually increased to 100 mg within the first week and maintained at that dosage for 8 weeks. In the combined treatment group, patients received sertraline as above and also underwent rTMS treatment using the VC-8000C magnetic stimulator.

During rTMS treatment, patients were positioned in a supine position to ensure comfort and stability throughout the procedure. The left dorsolateral prefrontal cortex (DLPFC) was selected as the stimulation site due to its significant role in mood regulation, particularly in patients with depression. Stimulating this area is known to improve emotional states by increasing cortical excitability and regional cerebral blood flow, thereby alleviating depressive symptoms^2^.

In our study, we utilized a low-frequency stimulation protocol at 1 Hz, employing continuous theta burst stimulation (cTBS). Each session consisted of 20 trains, with each train delivering 50 pulses and an inter-train interval of 5 s, resulting in a total of 1,000 pulses per session. Sessions lasted approximately 30 min and were conducted twice daily, with at least a 4-h interval between sessions to ensure adequate rest and minimize potential side effects. The treatment was administered 5 days per week over a period of 8 weeks.

### Clinical data collection

Clinical efficacy was evaluated based on changes in EPDS scores after 8 weeks. Cure was defined as a reduction in the EPDS score of 75% or more. Significant improvement was defined as a reduction between 50 and 74%. Moderate improvement was defined as a reduction between 25 and 49%. No significant change was defined as a reduction of less than 25%. The overall effectiveness rate was calculated as: (number of cured cases + number of significantly improved cases + number of moderately improved cases) divided by the total number of cases×100%^6,7^. Quality of life was assessed using the WHO Quality of Life-BREF (WHOQOL-BREF) questionnaire before treatment and 3 months post-treatment. Neurotransmitter levels, including serum 5-hydroxytryptamine (5-HT) and plasma norepinephrine (NE), as well as hormone levels of luteinizing hormone (LH), progesterone (P), estradiol (E2), and follicle-stimulating hormone (FSH), were measured before treatment and after 8 weeks.

### Statistical analyses

Normally distributed data are presented as mean±standard deviation (SD) and compared using the student's t-test. Within-group comparisons at different time points were conducted using the paired t-test. Categorical variables are presented as frequencies and analyzed using the chi-square test or Fisher's exact test. Logistic regression analysis was performed to identify factors associated with treatment effectiveness. Statistical analyses were conducted using SPSS version 22.0. p-values less than 0.05 were considered statistically significant.

## RESULTS

### Clinical characteristics of the patients

The study included 152 patients who met the inclusion and exclusion criteria, and they were divided into two groups: sertraline combined with rTMS (n=91) and sertraline alone (n=61). There were no significant differences between the groups in terms of age, marital status, obstetric history, mode of delivery, and educational level, indicating that the groups were comparable (all p>0.05; Table 1).

**Table 1:** Demographic and baseline characteristics of postpartum depression patients.

Characteristic		Sertraline+rTMS group (n=91)	Sertraline group (n=61)	p-value
Age (years), n (%)				0.564
	<30	55 (60.4)	34 (55.7)	
	≥30	36 (39.6)	27 (44.3)	
Marital status, n (%)				0.615
	Married	88 (96.7)	58 (95.1)	
	Divorced	3 (3.3)	3 (4.9)	
Gravidity, n (%)				0.818
	≤2	36 (39.6)	23 (37.7)	
	>2	55 (60.4)	38 (62.3)	
Parity, n (%)				0.268
	1 infant	70 (76.9)	42 (68.9)	
	≥2 infants	21 (23.1)	19 (31.1)	
Mode of delivery, n (%)				0.408
	Cesarean section	27 (29.7)	22 (36.1)	
	Vaginal delivery	64 (70.3)	39 (63.9)	
Educational level, n (%)				0.851
	High school and college	11 (12.1)	8 (13.1)	
	Bachelor's and above	80 (87.9)	53 (86.9)	
Neurotransmitter levels				
	5-HT (umol/L)	1.0±0.2	1.0±0.2	0.999
	NE (ng/L)	10.6±2.2	10.5±2.1	0.778
Quality of life				
	Physical function	58.7±7.8	58.3±6.9	0.74
	Psychological function	58.3±7.5	58.1±8.6	0.883
	Social relationships	59.1±8.1	59.4±7.9	0.821
	Environment	58.3±7.3	58.7±7.5	0.745
Estrogen levels				
	LH (U/L)	15.3±3.5	15.2±3.1	0.853
	FSH (U/L)	13.0±2.6	12.8±2.6	0.643
	P (nmol/L)	8.5±2.1	8.7±2.2	0.577
	E_2_ (pmol/L)	165.8±35.2	166.2±34.9	0.945

5-HT: 5-hydroxytryptamine; NE: plasma norepinephrine; LH: luteinizing hormone; FSH: follicle-stimulating hormone; P: progesterone; E_2_: estradiol.

### Comparison of treatment effectiveness

After 8 weeks, the sertraline plus rTMS group showed the following outcomes: 25.3% were cured, 53.8% showed significant improvement, 16.5% showed moderate improvement, and 4.4% had no significant change. In the sertraline group, 16.4% were cured, 44.3% showed significant improvement, 18.0% showed moderate improvement, and 21.3% had no significant change. The overall effectiveness rate was 95.6% for the combined treatment group, significantly higher than the 78.7% observed for the sertraline group (p=0.001).

### Factors affecting treatment effectiveness

Multivariate analyses revealed that patients aged under 30 years (adjusted OR 2.83; 95%CI 1.257–6.372; p=0.012), married patients (adjusted OR 1.82; 95%CI 1.046–3.166; p=0.034), and those receiving combined sertraline and rTMS treatment (adjusted OR 2.10; 95%CI 1.098–4.018; p=0.025) had significantly enhanced treatment efficacy for PPD (Table 2).

**Table 2:** Factors affecting the effectiveness of postpartum depression treatment.

Multivariate regression analysis				
Factors		Adjusted OR	95%CI	p-value
Age (years)				
	≥30	1 (Reference)	–	–
	<30	2.83	1.257–6.372	0.012
Marital status				
	Divorced	1 (Reference)	–	–
	Married	1.82	1.046–3.166	0.034
Gravidity				
	≤2	1 (Reference)	–	–
	>2	0.82	0.500–1.344	0.431
Parity				
	1	1 (Reference)	–	–
	≥2 infants	1.15	0.384–3.448	0.803
Mode of delivery				
	Cesarean section	1 (Reference)	–	–
	Vaginal delivery	0.72	0.103–5.050	0.741
Educational level				
	High school and college	1 (Reference)	–	–
	Bachelor's and above	1.05	0.857–1.286	0.637
Treatment group				
	Sertraline group	1 (Reference)	–	–
	Sertraline+rTMS group	2.10	1.098–4.018	0.025

OR: odds ratio; CI: confidence interval; rTMS: repetitive transcranial magnetic stimulation.

### Comparison of neurotransmitter levels

Baseline levels of 5-HT and NE were similar between the two groups. After 8 weeks of treatment, the combined treatment group had significantly higher levels of 5-HT (1.8±0.4 μmol/L) and NE (19.7±3.5 ng/L) compared to the sertraline group (5-HT: 1.4±0.3 μmol/L; NE: 15.2±2.6 ng/L; both p<0.001; Table 3).

**Table 3:** Post-treatment comparisons between the sertraline+repetitive transcranial magnetic stimulation group and the sertraline group.

Parameter		Sertraline+rTMS group (n=91)	Sertraline group (n=61)	p-value
Neurotransmitter levels				
	5-HT (umol/L)	1.8±0.4	1.4±0.3	<0.001
	NE (ng/L)	19.7±3.5	15.2±2.6	<0.001
Quality of life scores				
	Physical function	83.9±9.8	75.1±8.3	<0.001
	Psychological function	82.5±9.2	74.3±8.3	<0.001
	Social relationships	85.2±8.7	75.6±8.2	<0.001
	Environment	86.1±8.2	76.1±6.8	<0.001
Estrogen levels				
	LH (U/L)	10.2±1.9	12.7±2.3	<0.001
	FSH (U/L)	8.4±1.5	9.7±1.8	<0.001
	P (nmol/L)	14.3±3.2	11.3±2.7	<0.001
	E_2_ (pmol/L)	240.5±37.8	203.7±36.1	<0.001

rTMS: repetitive transcranial magnetic stimulation; 5-HT: 5-hydroxytryptamine; NE: plasma norepinephrine; LH: luteinizing hormone; FSH: follicle-stimulating hormone; P: progesterone; E_2_: estradiol.

### Comparisons of quality of life

At 3 months post-treatment, both groups showed improved quality of life scores across all dimensions compared to baseline. The combined treatment group had significantly higher scores than the sertraline group in all dimensions (p<0.001; Table 3).

### Comparison of estrogen levels

All patients were within 6 weeks postpartum and had not resumed menstruation during the study period. Baseline levels of LH, FSH, P, and E2 were similar between the groups. After 8 weeks of treatment, the combined treatment group had significantly lower LH and FSH levels and significantly higher P and E2 levels compared to the sertraline group (all p<0.001; Table 3).

### Comparison of adverse reactions

In the combined treatment group, adverse reactions included gastrointestinal discomfort (4.4%), fatigue (2.2%), insomnia (2.2%), and headaches (2.2%). In the sertraline group, adverse reactions included gastrointestinal discomfort (1.6%) and insomnia (3.3%). Notably, headache and fatigue, which are commonly known side effects of rTMS, were only reported in the combined treatment group. The overall rates of adverse reactions did not significantly differ between the groups (11.0 vs. 4.9%; p=0.190).

## DISCUSSION

In this study, the combined treatment of rTMS and sertraline was significantly more effective for patients with PPD compared to sertraline alone, highlighting the potential benefits of integrating rTMS with pharmacotherapy in managing PPD. Consistent with our findings, research by Avissar et al. investigated high-frequency rTMS targeting the left DLPFC and found significant reductions in patient scores on the Hamilton Depression Rating Scale (HAMD) compared to pre-treatment levels, demonstrating the effectiveness of rTMS in improving depressive symptoms^8^.

We identified several factors influencing therapeutic outcomes in PPD. Patients aged under 30 years, married women, and those receiving the combined treatment of sertraline with rTMS showed enhanced treatment effectiveness. Younger postpartum women may have a more favorable response due to better physical recovery and psychological resilience, facilitating swift adjustment in hormonal balance and psychological state, which may alleviate depressive symptoms. Married women experienced improved outcomes, possibly due to the social support inherent in marriage, including emotional backing and a network of social connections from spouses, family, and friends, which is crucial for mitigating depressive symptoms and enhancing the quality of life. Additionally, the combined therapy of sertraline and rTMS leverages the synergistic benefits of both pharmacological and physical treatments, significantly boosting effectiveness. This holistic approach may better modulate the brain's neurotransmitter systems and increase patient adherence and satisfaction with treatment, thereby enhancing the therapeutic efficacy for PPD^9^.

Our study demonstrates that the incidence of adverse reactions did not significantly differ between the group receiving combined sertraline and rTMS therapy and the group treated with sertraline alone (p=0.190), confirming the safety of rTMS as a treatment modality. Moreover, rTMS technology is advantageous due to its non-invasive, painless, and contact-free characteristics, coupled with a low incidence of adverse reactions^10,11^. These findings are consistent with previous studies indicating that rTMS is well-tolerated and has a favorable safety profile. These findings are consistent with previous studies indicating that rTMS is well-tolerated and has a favorable safety profile^12^. Similarly, Garcia et al. conducted a study involving nine PPD patients treated with rTMS and observed significant reductions in scores on various scales, including the HAMD, EPDS, Inventory of Depressive Symptomatology-Self-Rated (IDS-SR), and Clinical Global Impression-Severity (CGI-S) after just 2 weeks^13^. Notably, seven of these patients maintained therapeutic benefits for at least 180 days, further corroborating the safety and long-term efficacy of rTMS treatment.

Fluctuations in neurotransmitter levels, particularly 5-HT and NE, play crucial roles in the development and progression of PPD. Fluctuations in neurotransmitter levels, particularly serotonin (5-HT) and norepinephrine (NE), play crucial roles in the development and progression of PPD^1^. NE influences mood, emotions, and behavior, and reduced levels are associated with exacerbated depressive symptoms. Therefore, managing 5-HT and NE levels is essential in PPD treatment^14^. Our findings reveal that after an 8-week treatment period, patients receiving combined sertraline and rTMS therapy exhibited a significantly higher overall effectiveness rate than those treated with sertraline alone. Notably, 5-HT and NE levels were significantly elevated in the combined treatment group compared to the sertraline-only group (p<0.001).

These results suggest that while sertraline alone is effective in treating PPD, its combination with rTMS enhances therapeutic outcomes and boosts neurotransmitter levels. This enhancement can be attributed to the effects of rTMS as a neuro-electrophysiological technique. rTMS modulates neuronal activity in the brain by applying magnetic pulses to specific cortical areas, such as the DLPFC, which is involved in mood regulation. This modulation may enhance the excitability of the targeted area and interconnected regions, improving neurotransmitter function and thus enhancing treatment efficacy^3^.

Estrogen plays a crucial role in the development and progression of PPD. During late pregnancy, estrogen levels are elevated but rapidly decline following childbirth, leading to significant hormonal fluctuations that may cause neuroendocrine dysfunction and increase the risk of PPD^15^. Therefore, stabilizing estrogen levels is essential in PPD treatment. Our study found that after 8 weeks of combined sertraline and rTMS therapy, patients showed notable improvements in physical and psychological functions, social relationships, and environmental adaptability, surpassing those treated with sertraline alone. Furthermore, the combined therapy group exhibited significant reductions in LH and FSH levels and increases in P and E2 levels compared to the sertraline-only group (p<0.001). These results indicate that the combined sertraline and rTMS treatment not only positively affects estrogen levels but also substantially improves patients’ overall quality of life.

## CONCLUSION

Combining sertraline with rTMS for the treatment of PPD significantly enhances clinical outcomes by improving neurotransmitter and hormone levels and elevating the quality of life without increasing adverse reactions. This integrated approach leverages the benefits of both pharmacological and physical therapies, offering a comprehensive and effective treatment modality for PPD.
